# Adaptive Mechanical Metamaterials with On‐Demand Binary Local Modulus for Embodied Intelligence

**DOI:** 10.1002/advs.202509675

**Published:** 2025-08-26

**Authors:** Richard J. Nash, Yunzheng Yang, Yaning Li

**Affiliations:** ^1^ Department of Mechanical and Industrial Engineering Northeastern University Boston MA 02215 USA

**Keywords:** adaptive materials, additive manufacturing, embodied intelligence, mechanical metamaterials, on‐demand

## Abstract

Biological materials in nature are inherently adaptive, evolving through continuous interaction with their environment. Achieving such adaptability and self‐optimization in artificial materials remains a major challenge. In this work, a simple yet robust mechanism is introduced that enables instantaneous changes in local stiffness components in response to strain. This is realized by designing binary meta‐capsules with two discrete states 0 and 1, each corresponding to a different modulus in one direction. These strain‐responsive capsules switch states based on applied deformation, serving as the building blocks for a new class of adaptive mechanical metamaterials (AMMs). Computational tools are developed to guide the design, and selected structures are fabricated via multi‐material polymer jetting. Mechanical experiments, including compression and indentation tests, confirm the functionality of the AMMs. Because the stiffness change in each meta‐capsule is reversible, the material can reconfigure itself after loading‐unloading cycle. This enables AMMs to dynamically adjust their local properties based on external loads and/or constraints, effectively “reprogramming” or redesigning themselves post‐fabrication, paving the way for transforming 3D/4D printing into adaptive, “infinity‐D” printing.

## Graphical abstract

Inspired by nature's adaptivity, this work presents a new class of mechanical metamaterials that can reversibly switch local stiffness in response to local strain. Using strain‐triggered binary meta‐capsules as building blocks, these materials dynamically reconfigure their properties under load, enabling embodied intelligence and paving the way toward truly adaptive, reprogrammable “infinity‐D” printed structures.

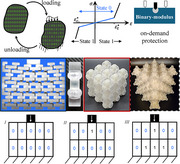

## 1 Introduction

Conventional engineering materials are typically manufactured through uniform, static processes and possess fixed properties that degrade irreversibly over time. In stark contrast, biological materials found in nature exhibit remarkable adaptability, continuously evolving in response to environmental cues across multiple time scales. This adaptability is a product of their heterogeneous architectures and dynamic structure‐property relationships. For instance, living bone and wood remodel their microstructure, density, and stiffness to optimize load‐bearing capacity;^[^ ^1^, ^2^, ^3^, ^4^ ^]^ pinecones passively actuate in response to humidity to enable seed dispersal;^[^ ^5^, ^6^ ^]^ cephalopods rapidly alter their skin's appearance for camouflage;^[^ ^7^, ^8^ ^]^ and cells and muscles exhibit stress‐stiffening and anisotropic behaviors that enhance functionality and resilience.^[^ ^9^, ^10^, ^11^ ^]^ These examples demonstrate how natural systems achieve complex, real‐time adaptation through embedded material intelligence, whether over months (bone remodeling),^[^ ^12^, ^13^, ^14^, ^15^, ^16^, ^17^, ^18^, ^19^, ^20^, ^21^, ^22^ ^]^ hours (pinecone actuation),^[^ ^5^, ^6^ ^]^ or mere seconds (cephalopod camouflage).^[^ ^7^, ^8^ ^]^ Despite growing interest, replicating such adaptive capabilities in synthetic materials remains a significant challenge. This motivates the development of engineered systems that can emulate biological adaptability through embedded, stimuli‐responsive design. The research presented here contributes to this effort by introducing novel materials capable of programmable, on‐demand mechanical responses, bringing us closer to creating artificial materials that not only perform but evolve, adapt, and self‐optimize in response to their environment.

Biological adaptation has been investigated by biologists, computer scientists, and engineers for various kinds of systems.^[^ ^2^, ^3^, ^4^, ^9^, ^10^, ^11^, ^23^, ^24^, ^25^, ^26^, ^27^, ^28^, ^29^, ^30^, ^31^, ^32^ ^]^ Material engineers have made great progress in developing engineering adaptive materials in the last decades. For synthetic adaptive materials, the common strategy for achieving adaptivity is to make their inherent mechanical, or optical, or chemical^[^ ^5^, ^13^ ^]^ properties be more dynamic, responsive, and stimuli‐sensitive. For example, self‐strengthening materials have made great progress in harnessing mechanical energy for constructive processes via mechanophores^[^ ^33^, ^34^, ^35^, ^36^ ^]^ or mechanoradicals.^[^ ^37^, ^38^ ^]^ To trigger the self‐strengthening through this process requires a large strain from shear or axial force, and often once triggered, being non‐reversible, and therefore non‐reusable.^[^ ^39^, ^40^ ^]^ A composite material was developed that adapts its modulus in response to applied force, duration, and the frequency of mechanical agitation,^[^ ^13^ ^]^ enabling adaptivity under relatively small strains. Chemical stiffening have been shown via hydration of aerogel substances^[^ ^33^ ^]^ resulting in permanent change due to alteration in the microstructure. Also, reversible chemical stiffening are shown in hydrogels and liquid crystal elastomers.^[^ ^34^, ^35^ ^]^ Ultrafast thermal stiffening has been achieved by using a unique combination of thermal‐responsive resins,^[^ ^36^ ^]^ and this method was proven to be both reversible and displayed efficient vibration‐damping properties.^[^ ^37^, ^38^ ^]^ Other methods such as electrically or magnetically induced, or in response to light has been shown as well.^[^ ^39^ ^]^

While traditional adaptive materials rely on either chemical (e.g., pH, salinity) or physical (e.g., temperature, light) stimuli to trigger responses in relatively homogeneous systems,^[^ ^41^, ^42^ ^]^ emerging advances in 3D printing offer new opportunities to integrate structural logic into architected materials. These capabilities enable mechanical signal processing and adaptation through the principles of mechanical metamaterials.^[^ ^43^ ^]^ Significant progress has been made in engineering mechanical metamaterials with pre‐programmed, tunable, and enhanced functionalities, such as negative Poisson's ratio,^[^ ^12^, ^13^, ^14^, ^15^ ^]^ programmable stiffness,^[^ ^44^, ^45^, ^46^ ^]^ improved strength and toughness,^[^ ^18^, ^20^, ^21^ ^]^ tailored thermal expansion,^[^ ^23^, ^24^, ^25^, ^26^ ^]^ and increased flexibility.^[^ ^27^, ^28^ ^]^ For example, relevant work on programmable mechanical metamaterials include programmable Quasi‐Zero‐Stiffness metamaterials,^[^ ^44^, ^45^ ^]^ knitted materials,^[^ ^46^ ^]^ and phase transforming cellular materials.^[^ ^47^ ^]^ Recently, mesoscale design strategies involving snap‐through instabilities and bistable geometries have enabled logic‐like mechanical behavior in structured solids.^[^ ^47^, ^48^, ^49^, ^50^, ^51^ ^]^ However, due to the sensitivity and nonlinearity of instability‐driven mechanisms, achieving robust, repeatable,^[^ ^52^ ^]^ and tunable adaptation remains a major challenge, particularly under small‐strain conditions. To address this challenge, we introduce a new class of Adaptive Mechanical Metamaterials (AMMs) composed of strain‐responsive binary meta‐capsules capable of instantaneous and reversible modulus switching.

As shown in **Table** **1**, existing adaptive material systems relying on external stimuli such as temperature, magnetic fields, or global mechanical actuation offer continuous but relatively slow or material‐limited tunability. In contrast, our strain‐triggered metamaterial enables localized, rapid, and reversible binary stiffness switching through purely mechanical input, without requiring environmental changes or external actuation fields. This geometrically encoded adaptivity provides a distinct mechanism for achieving programmable and repeatable mechanical responses in architected materials.

**Table 1:** Comparison of representative adaptive material (AM) systems.

System type	Triggering mechanism	Reversibility	Responsiveness	Tunability
Thermally responsive AM	Temperature	Partial	Slow/Moderate	Continuous
Magneto‐active AM	Magnetic field	High	Moderate/Fast	Continuous
Origami/kirigami metamaterials	Mechanical actuation	Partial	Moderate	Structural, multistable
Swelling‐induced architectures	Solvent or pH	Partial	Slow	Continuous
This work: Strain‐triggered contact metamaterial	Local mechanical strain (contact)	High	Fast	Binary, spatially programmable

In this investigation, two fundamental mechanisms are explored to realize this binary stiffness behavior. Design variants include both 1D and 3D architectures, fabricated using multi‐material 3D printing. A comprehensive design‐to‐evaluation framework is developed, integrating adaptive finite element (FE) modeling with experimental characterization. The adaptive response of the AMM is both reversible and repeatable, allowing the material to dynamically reconfigure its mechanical properties under various loading scenarios. Following unloading, the material can “restart”, offering a route toward next‐generation reprogrammable architectures^[^ ^47^, ^48^ ^]^ with embodied physical intelligence and logic‐like mechanical behavior.^[^ ^49^, ^50^ ^]^

## 2 The Concept of AMMs with On‐Demand Binary Local Modulus

Embodied intelligence refers to a system's ability to adapt and respond to external stimuli through its physical structure and material properties, without centralized control. In synthetic materials, this concept is increasingly realized through architected systems that encode functional responses, such as shape change or stiffness modulation, directly into their geometry or composition. The adaptive mechanical metamaterials (AMMs) introduced in this work embody this principle by integrating strain‐responsive meta‐capsules with on‐demand binary stiffness. These local units switch between soft and stiff states based solely on mechanical input, enabling distributed autonomous adaptation across the structure. This decentralized responsiveness allows the material to dynamically adjust its mechanical behavior in real time, forming a foundation for programmable, self‐regulating systems with embedded intelligence.

**Figure 1:**
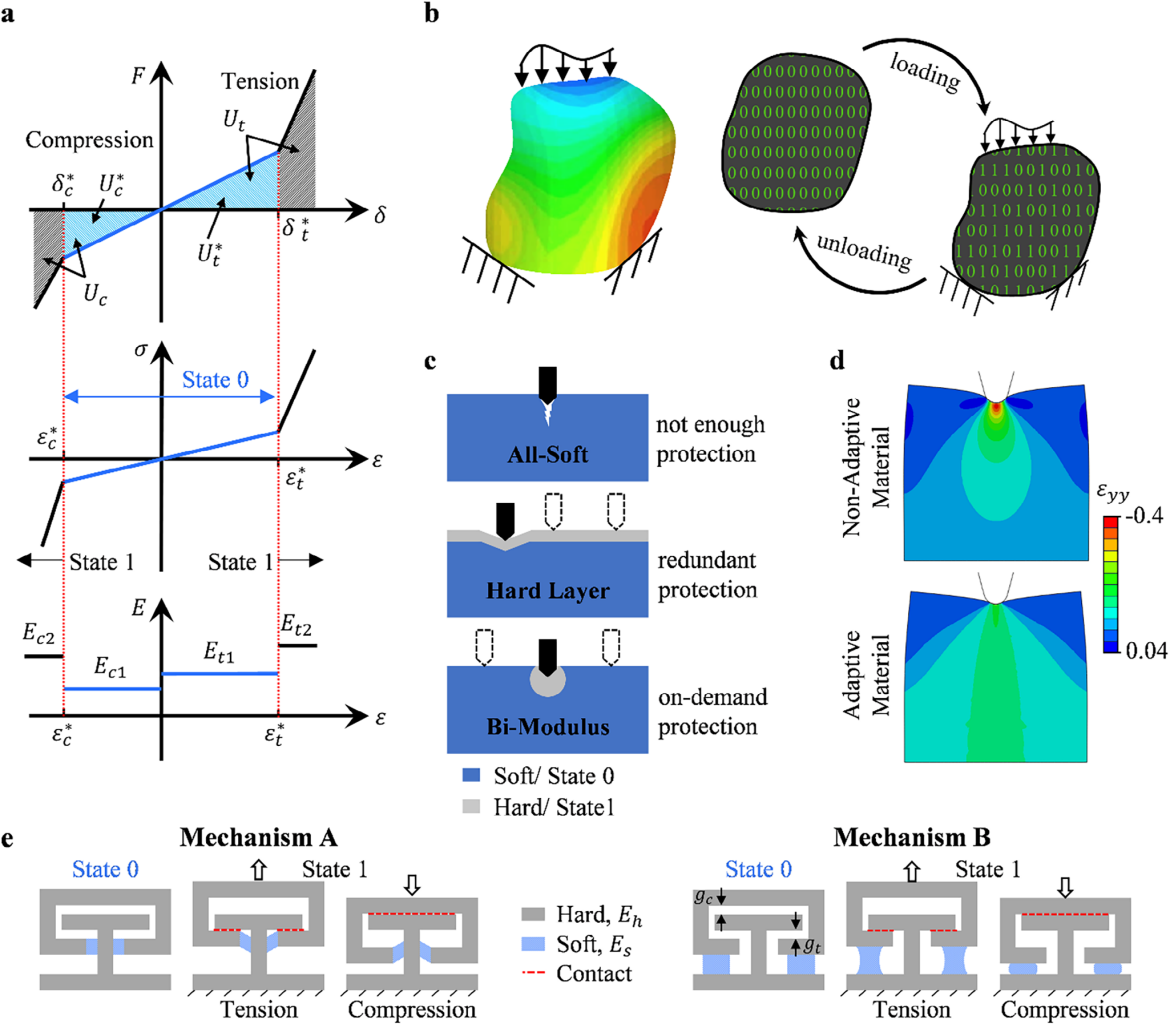
Conceptual formulation and design schematics of the AMMs. a) Representative load‐displacement, stress‐strain, and elastic modulus‐strain curves for a typical bi‐modulus material, depicting the initial moduli, secondary moduli, critical deformations, and States 0 and 1 represented by the blue and black regions respectively, b) a schematic showing the strain contours on a body due to varying loading and boundary conditions (left) and the local binary response, switching between States 0 and 1, c) schematics comparing three different design strategy, an all soft (top), a soft material with hard layer on top (middle), and the AMMs (bottom) where the blue and grey regions represent soft and hard material phases, respectively, d) y‐strain contours are shown for a non‐ adaptive material (top) and for an adaptive material (bottom) for the same indentation depth, e) the schematics of a 1D bi‐modulus meta‐capsules in State 0, and in State 1 under tension and compression for both Mechanism A and Mechanism B.

The AMMs studied here exhibit a unique material property in which the materials instantaneously change local elastic modulus upon reaching a critical criterion. Conventional material can sometimes exhibit stiffening or softening behavior throughout deformation via nonlinear deformation at microscale, inelastic material response, and various other reasons, however the AMMs discussed are able to achieve this in the elastic regime under small deformation, i.e., the change is reversible. This behavior is referred to as “bi‐modulus” and is defined here has having an initial elastic modulus, *E*_1_, and after a critical criterion has been met, the material properties change to the secondary elastic modulus, *E*_2_. In general, a critical criterion for triggering the change in stiffness can vary, but here, the criterion for change in modulus is defined by a critical deformation, which can be either the critical displacement, δ*, or the critical strain, ε*. The concept of bi‐modulus behavior is illustrated in **Figure** **1**.

The overall force‐displacement, stress‐strain, and elastic modulus‐strain response of a bi‐modulus material is depicted in Figure 1a, where *F* represents the applied force and δ represents the global displacement. Here, it is shown that each property of a bi‐modulus material, i.e. critical deformation and elastic modulus, may have different tunable values in tension and in compression, resulting in corresponding subscripts of *t* and *c* respectively. The effective stress‐strain and load‐displacement responses have two distinct regions of deformation separated by the vertical red dashed lines in Figure 1a. In the blue region between the red dashed lines is the initial deformation stage State 0, where ε ≤ ε*, δ ≤ δ*, stiffness matrix [*E*] = [*E*_1_], and in the black regions outside the red dashed lines is the secondary deformation stage State 1, where ε ≥ ε*,  δ ≥ δ*, and [*E*] = [*E*_2_]. The elastic modulus‐strain curves depict how for an ideal bi‐modulus material, the change from initial to secondary modulus will be instantaneous at the critical deformation, resulting in the following key stress‐strain formulation:

(1) $$\sigma =\left\{\begin{array}{cc} {}[E_{1}]\varepsilon , & \varepsilon \leq \varepsilon^{*}\\ {}[E_{1}]\varepsilon^{*}+[E_{2}]\left(\varepsilon -\varepsilon^{*}\right), & \varepsilon \geq \varepsilon^{*} \end{array}\right.,$$

To numerically implement this unique binary constitutive mode, a user‐defined material (UMAT) subroutine in ABAQUS is developed based on Equation (1). Details of the approach are provided in Section SB (Supporting Information). For elastic deformation of a 1D bi‐modulus material, the blue area under the load‐displacement curves up until δ* is the critical strain energy required to change state, U*, Equation (2). The total area under the force‐displacement curve is the strain energy, *U*, required to deform the material to any given displacement, δ, given by Equation (3), where *A* is the cross‐sectional area of the material normal to the loading direction, and *L* is the initial length of the sample.

(2) $$U^{*}=\frac{1}{2}\frac{E_{1}A\delta^{*2}}{L}$$

(3) $$U=\left\{\begin{array}{cc} \frac{1}{2}\frac{E_{1}A\delta^{2}}{L}, & \delta \leq \delta^{*}\\ \frac{A}{2L}\left(\left(2\delta^{*}\delta -\delta^{*2}\right)E_{1}+\left(\delta^{2}-2\delta^{*}\delta +\delta^{*2}\right)E_{2}\right), & \delta \geq \delta^{*} \end{array}\right.$$

For 1D bi‐modulus material, the blue area under the stress–strain curves up until ε* is the critical strain energy density is required to change state, $U_{d}^{*}$, Equation (4). The total area under the stress–strain curve is the strain energy density, *U_d_*, required to deform the material to any given ε, Equation (5).

(4) $$U_{d}^{*}=\frac{1}{2}E_{1}{\varepsilon^{*}}^{2},$$

(5) $$U_{d}=\left\{\begin{array}{cc} \frac{1}{2}E_{1}\varepsilon^{2}, & \varepsilon \leq \varepsilon^{*}\\ \frac{1}{2}E_{1}\left(2\varepsilon^{*}\varepsilon -{\varepsilon^{*}}^{2}\right)+\frac{1}{2}E_{2}{\left(\varepsilon -\varepsilon^{*}\right)}^{2}=\left(\frac{1}{2}{\varepsilon^{*}}^{2}-\varepsilon^{*}\varepsilon \right)\left(E_{2}-E_{1}\right)+\frac{1}{2}\varepsilon^{2}E_{2}, & \varepsilon \geq \varepsilon^{*} \end{array}\right.$$

Because the deformation is reversible, making it possible for the material to switch from State 0 to State 1 and back again as necessary, using this material in a continuum will enable it to adapt to the various load cases it may experience, stiffening and strengthening the material in only the locations it needs to and leaving the rest compliant. The continuum can be thought of as a binary system of 0's and 1's in which each material point is given a value of 0 if it is in State 0, or 1 if it is in State 1. Visualization of which material points change state during the loading and unloading processes now becomes possible as depicted in Figure 1b.

The benefits of bi‐modulus AMMs are easily exhibited in systems experiencing high stress and strain concentrations, depicted by the three indentations schematics in Figure 1c. An all‐soft material might not provide the necessary protection against such a load, while the conventional hard layer strategy provides redundant protection by protecting everywhere at the cost of its compliance and weight. The bi‐modulus sample illustrates that only the areas in need are reinforced dynamically, providing on‐demand protection and maintaining the compliance of the rest of the sample.

Figure 1d shows the y‐strain contours for finite element (FE) indentation simulations of a non‐adaptive (NA) material using a linear elastic (LE) material model and for an adaptive material, via a user defined material (UMAT) constitutive model (Equation 1), at the same level of indentation, illustrating how the bi‐modulus adaptive material can activate and utilize more of the material to resist the concentrated load, resulting in stiffening under the indenter tip but leaving the unaffected material unchanged.

Usually, to obtain a bi‐modulus material, either two different deformation mechanisms or two different material phases are required, functioning such that one mechanism or phase is responsible for the first region of deformation, State 0, while the other is responsible for the second region of deformation, State 1. The materials designed here utilize two material phases to achieve bi‐modulus behavior, a hard phase with Young's Modulus *E_h_*, and a soft phase with Young's Modulus *E_s_*. Starting with a 1D design concept, the two‐phase bi‐modulus materials designed here are created such that the soft material is much softer than the hard, and that the soft material is deforming and responsible for load bearing before ε*, while the hard material is responsible for after ε*.

1D meta‐capsules are designed such that their critical deformation induces contact between two or more hard phase surfaces, resulting in increased modulus in responding to load in one direction, the concept of which is shown in Figure 1e. The deformation of the soft phase under global tension or compression is defined as either shearing in the soft layer, Mechanism A, or tension in the soft layer, Mechanism B. The prescribed initial gaps for compressive and tensile deformation, *g_c_* and *g_t_*, define the critical deformation for these cases because when the gaps shrink to zero due to deformation of the soft phase, the material changes from State 0 to State 1. Section SA (Supporting Information) describes how the prescribed initial gaps are used to determine the critical strain. This means that out of all the energy put into the material to deform it, U* is a set amount that will go into only the soft phase. As long as the material is designed such that U* is less than the energy required to damage the soft phase, it is expected that failure will eventually occur in the hard phase first.

## 3 3D Printed 1D Bi‐Modulus AMM Designs

### 3.1 Designs and Uniaxial Compression Experiments

The first AMM discussed utilizes shearing mechanism A and consists of an offset arrangement of hard phase “I” pieces joined to their neighboring pieces via smaller sections of soft phase. Here, State 0 consists of the shearing of the soft phase and is followed by the contact of neighboring “I” pieces in State 1, resulting in bi‐modulus behavior along one direction, called the I‐Beam design. A detailed description of the designs and geometry is provided in Section SA (Supporting Information), along with a schematic of the representative volume element (RVE) labeling the relevant geometric parameters in Figure S1 (Supporting Information), and their prescribed values in Table S1 (Supporting Information).

**Figure 2:**
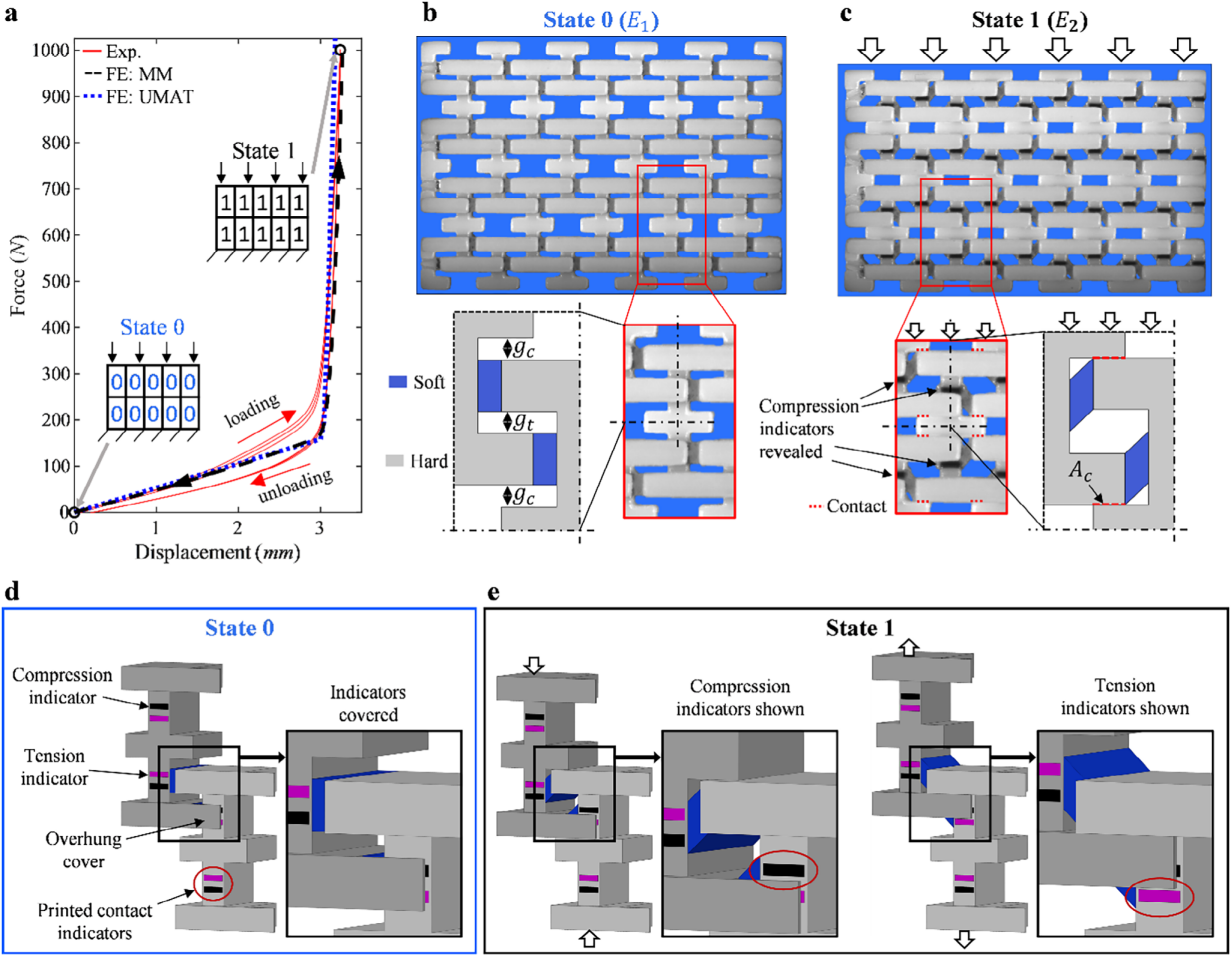
The schematics, experimental, and simulation results for the compression of the bi‐modulus material design with indicators. a) The force‐displacement results for the experimental and FE results (both the modeled microstructure and UMAT model) for three cycles of uniaxial compression where the initial State 0 and State 1 are marked, and their binary representations are shown, b,c) experimental images of the marked location for State 0 and State 1 respectively (top), along with the experiential image of a RVE and a schematic of a quarter of the RVE labeled with relevant geometric parameters, where the blue and grey materials represent the soft and hard material phases respective, and the red dazed lines indicate contact, and the compression indicators are visible for State 1, d,e) the schematic of the surface alterations made of the bi‐modulus design to visualize the state changes during experimental deformation for State 0 and State 1 respectively, where the black marker is the compression indicator and the magenta marker is the tension indicator.

A 2 RVE tall by 5 RVE wide sample is manufactured for cyclic compression experiments and FE simulations are run on both the modeled microstructure (MM) and with the UMAT model, the results of which are shown in the force‐displacement curve in **Figure** **2**a, where the initial and final configurations are marked, representing State 0 and State 1 respectively, and their binary representations are shown. Both experimental and FE results correlate well with each other and show a clear bi‐modulus behavior brought about by a critical activation strain. As the soft phase material is a viscoelastic material, there is some hysteresis observed during the deformation in State 0 for the experimental sample, however this behavior is not captured by its material model for static FE simulations. It should also be noted that this viscoelastic property results in a small residual displacement after unloading which is allowed to fully recover before the next cycle of loading is started. Figure 2b,c shows the experimental images of the marked states, each with a close‐up of a single RVE and a schematic of a quarter of the RVE possessing mirror symmetry on each edge. For compressive deformation, when gap *g_c_* goes to 0, neighboring hard phases touch over a contact area of *A_c_*, indicated by the red dashed lines.

To visualize these states experimentally, surface alterations are made to the core design in which tension (magenta) and compression (black) indicators, and an overhanging section are added to the top surface of the design. The SolidWorks models are shown for two neighboring “I” pieces (grey), their connecting soft layer (blue), and surface alterations for both State 0, Figure 2d, and State 1, Figure 2e. In the default State 0, the overhangs cover the indicators on neighboring “I” pieces and upon deformation the overhangs move to reveal the indicators and are designed to be fully reveled in State 1. For this case of compressive loading, the black bi‐modulus indicators are revealed, and the RVE is considered switched from State 0 to State 1 when all six bi‐modulus indicators present are fully revealed as seen in Figure 2c (two full indicators in the middle and four half‐indicators on the edges).

### 3.2 Cyclic Indentation Experiments

**Figure 3:**
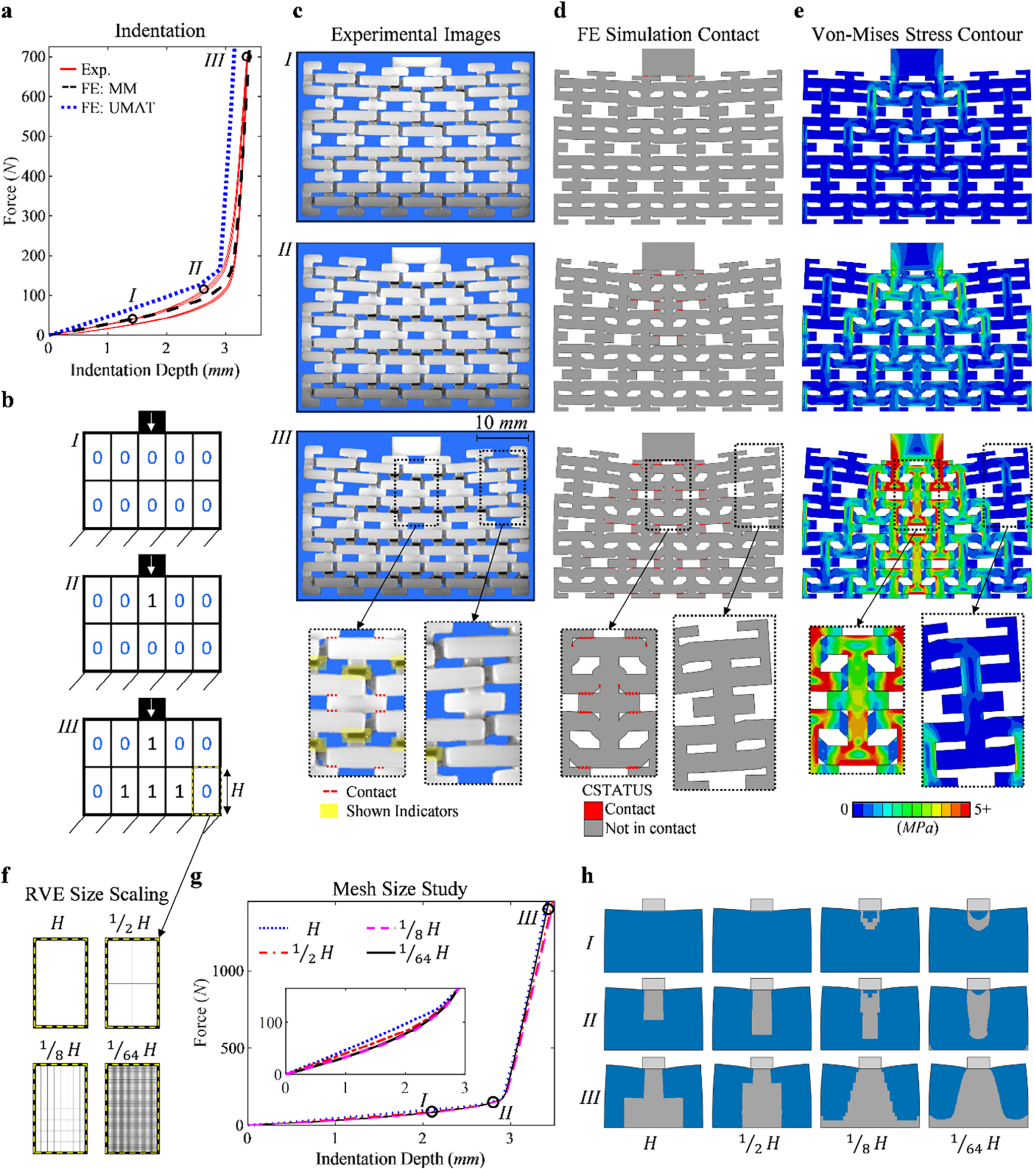
The experimental and simulation results for the indentation of the bi‐modulus material design with indicators. a) The experimental and FE (both the modeled microstructure and UMAT model) force‐indentation depth results for three cycles of indentation where three locations during the deformation are marked and analyzed, b) the binary representations for each of the marked locations, c–e) experimental sample images, FE simulation visualizing the contact locations, and FE Von‐Mises stress contours respectively, for each marked location followed by close ups of the top middle and top right RVEs for the final marked location, f) the mesh sizes used in the mesh size study performed for the UMAT simulation models, g) the force‐indentation depth results for the mesh size study, h) the binary representation of the states of each element and the shown marked locations with blue and grey representing State 0 and State 1 respectively.

Cyclic indentation experiments and both MM and UMAT FE simulations are now performed on the I‐Beam design to observe how the AMM is able to adapt to loadings exhibiting stress or strain concentrations. The force‐indentation depth results are shown in **Figure** **3**a, in which stages *I–III* are marked representing small deformation before any state change, the point where the first cell switches state, the maximum deformation seen in the sample, respectively. The binary representation of each stage is shown in Figure 3b, experimental images of the sample is shown in Figure 3c, the MM FE simulation in which contact has been visually displayed by ABAQUS is shown in Figure 3d, and the MM FE simulation of the Von‐Mises stress contours is shown in Figure 3e.

Stage *I* shows that some partial uncovering of bi‐modulus indicators is present, but none have been fully revealed, and the only contact seen in the FE model is between the indentation block and the sample where a small stress concentration can also be seen. In stage *II* the top middle RVE has reached its critical deformation and switched form state 0 to State 1, shown by the six fully revealed indicators and by the contact output, where again contact at all six possible locations within the RVE indicates state change. From both visualization from experiments and contact in the FE simulation, stage *III* shows that all cells aligned under the indenter have been switched, along with the two neighboring cells in the bottom. This results in the effective hardening in the region under the tip and load bearing is now happening the hard phase, which in turn results in a large increase in the force, stresses, and the materials resistance to the indentation load. Close‐up views of the top middle and the top right cells for stage *III* clearly show the different states present in the material via the bi‐modulus indicators, contact in the FE model, and larger stresses.

The matching FE and experimental results verify that the I‐Beam design and its bi‐modulus behavior is accurately captured from the FE models and allows for the examination of more advanced AMM systems via FE simulations, ones that are not possible for the current scale of additive manufacturing. As the UMAT method models the entire RVE as a single element, an initial mesh size study is performed, in which the amount of RVEs is increased from the initial size used in 2 × 5 modeling to 1/2, 1/8, and 1/64 of the original size, shown in Figure 3f. The force‐indentation depth result are shown in Figure 3g, and although there are little changes seen in the resulting curves, Figure 3h shows the simulation's visualized state results at the marked locations *I–III* depicting how the changes in state from State 0 (blue) to State 1 (grey) depend on the mesh size and converge with an in increasing mesh density.

### 3.3 Comparison Between Adaptive vs Non‐Adaptive Behaviors via FE Simulations

**Figure 4:**
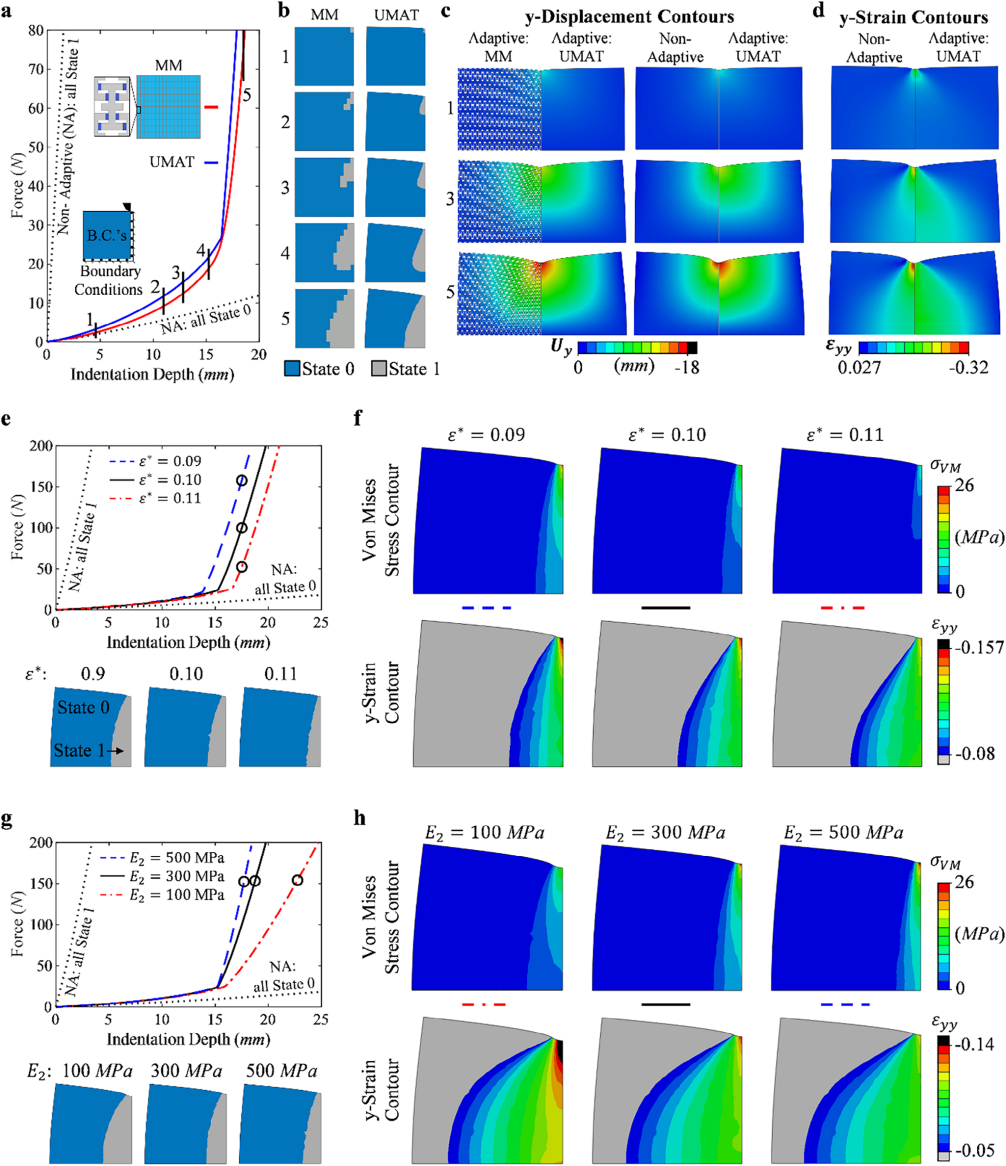
A numerical analysis of the adaptive material simulations compared to non‐adaptive material models. a) The FE force‐indentation depth results for the modeled microstructure (MM), UMAT, and non‐adaptive (NA) cases where the material is all State 0 (blue) and all State 1 (grey), along with the boundary conditions (BCs) of the models used, b) the visualization of states in each element during the indentation at the marked locations in a) for both the MM and UMAT simulations, c) comparisons of the displacement contours at locations 1, 3, and 5 for the MM and UMAT, showing the identical behavior, and between the UMAT and NA models to show the difference between adaptive and non‐adaptive materials, d) comparisons of the y‐strain contours for the non‐adaptive material models and the adaptive material models, e) the force‐indentation depth results for the parametric study in which the critical strain is varied (top) and the resulting state visualization at the same indentation depths marked in the curves (bottom), f) the Von‐Mises stress contour and the y‐strain contour at the marked locations in e) for each critical strain, g) the force‐indentation depth results for the parametric study in which the secondary modulus is varied (top) and the resulting state visualization at the same force marked in the curves (bottom), h) the Von‐Mises stress contour and the y‐strain contour at the marked locations in g) for each critical strain.

To better understand how an AMM adapts to stress concentrations, a larger amount of RVEs within a single sample is ideal, i.e. more RVE's than the previous 2 × 5 RVE design. However, in lieu of manufacturing a very large sample, this study is performed solely numerically, as it has been shown above that the MM and UMAT models accurately simulate the behavior of the experimental sample. The indentation of a 150 mm tall by 150.45 mm wide simply supported sample with mirror symmetry about the right edge is simulated for four cases. Cases 1 and 2 are the adaptive materials where case 1 is the MM having an identical RVE as before and is 17 RVEs wide and 10 RVEs tall, while case 2 is a finely meshed continuum of the UMAT model. Cases 3 and 4 are the non‐adaptive materials and utilize the same fine mesh but are non‐adaptive (NA) and have LE material models with equivalent stiffnesses of State 0 and State 1. The force‐indentation depth response for each, along with the prescribed boundary conditions (BCs) is shown in **Figure** **4**a.

The visualization of state for the RVEs for MM and in the elements for the UMAT model is shown in Figure 4b for each location marked 1–5. The MM visualization is created by locating the regions of contact within the model and manually coloring the individual RVE, resulting in an undeformed visualization, while the UMAT state visualization is automatically generated via ABAQUS, and is able to capture the overall sample deformation. These representations of the current states of the material exhibit how the material is dynamically reprogramming itself for the current level of external loading. To further illustrate the similarities between the two, Figure 4c shows the Y‐displacement contour comparison of MM and UMAT (left). The force‐indentation depth, state visualization, and deformation of these simulations at each step shown provide confirmation that the UMAT model sufficiently represents both the MM model and the experimental samples. It is now possible for a direct comparison of adaptive and NA materials via the UMAT and LE models respectively. Figure 4c (right) compares the Y‐displacement contours for each, highlighting how for the same level of indentation the non‐adaptive designs experience the same maximum displacement, however a much smaller quantity of material is deformed. The y‐strain contour comparison in Figure 4d further highlights this behavior, and additionally shows that the adaptive material reduces the strain concentration that occurs directly under the indenter, while deforming a larger quantity of material to resist it. These differences between the adaptive and non‐adaptive materials are shown to increase with increasing levels of indentation.

A parametric study is performed with the UMAT model to observe the effects that different key parameters, the critical deformation and secondary modulus, have on the adaptive materials response to this indentation load. Figure 4e shows the results for a varying critical deformation and shows the visualization of states for the locations marked, corresponding to the same displacement for each. Figure 4f displays the Von Mises stress contours and y‐strain contours at the marked locations, showing how the overall stress, stress concentration under the indenter tip, and strain concentration under the indenter tip, reduce with increasing critical deformation. The amount of material deformed however increases with and increasing critical deformation.

The secondary modulus is varied in Figure 4g,h while the critical deformation is held constant, and the marked locations this time compare cases at the same indentation load. An increasing secondary modulus results in less elements changing state, larger stresses and stress concentrations underneath the indenter tip, and a smaller area of stress distribution. This also results in a significant reduction in strain concentration underneath the indenter tip without affecting the amount of material utilized to resist deformation. By specifically tuning these critical parameters, the materials can be programed for general applications such as efficiently resisting impacts, or more specific applications such as protecting against point loads.

## 4 3D AMMs

### 4.1 1D Bi‐Modulus Meta‐Capsule

**Figure 5:**
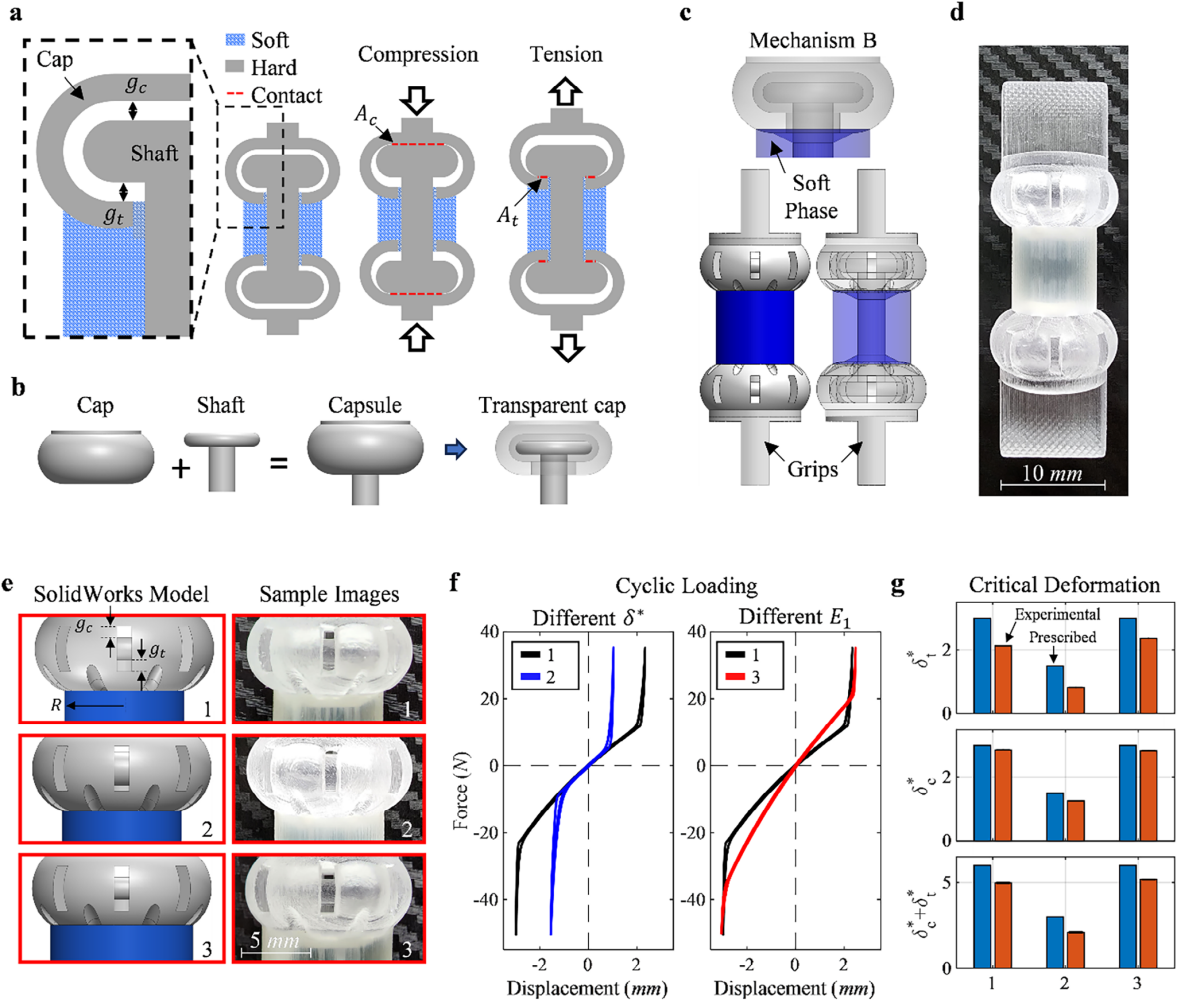
The schematics and experimental results for a 1D bi‐modulus material that functions under both tension and compression. a) The schematics of a capsule comprised of a hard and soft phase, exhibiting bi‐modulus behavior under both tension and compression where the blue area represent the soft material, grey area represents the hard materials, the red dashed lines show areas of contact, b) a schematic of the individual hard phase components of the generic bi‐modulus capsule and the transparent assembly, c) the SolidWorks model of the bi‐modulus capsule utilizing Mechanism B, with the added grips for mechanical testing of the design shown with a solid and transparent view, d) an image of the B2 experimental sample, e) close up views of the SolidWorks models and experimental samples for Designs B1–B3, f) the experimental force‐displacement results for all three cycles of loading compared between B1 and B2 (left) and B1 and B3 (right), g) a comparison of the prescribed critical deformation for each sample with their experimental values.

A 1D bi‐modulus capsule is designed as a proof‐of‐concept starting point for the 3D design discussed next, and to demonstrate the tunability of the initial modulus and the critical deformation. **Figure** **5**a shows the schematic for an arbitrary capsule design consisting of a hard phase “I” shaped central shaft with a hard phase cap completely encompassing, but not touching, each end of the shaft. A soft phase (not necessarily to be a solid continuum) is attached to both the shaft and each cap as well, allowing for movement of the caps via deformation of the soft phase. Like the I‐Beam design, when corresponding gaps *g_t_* or *g_c_* go to zero for tension or compression, the capsule switches from State 0 to State 1.

For simplicity the soft phase is made solid, and a solid revolution of the schematic in Figure 5a about its center is performed and grips are added to the caps for mechanical testing, to create the 1D Capsule design. Figure 5b shows the composition of the hard phases (the caps and the inner shaft), along with a transparent view of the assembly. Figure 5c illustrates the capsule design utilizing deformation Mechanism B with a solid and a transparent SolidWorks model of the sample where the soft phase is colored blue and hard phase is colored grey. The slits seen in the caps are for post‐manufacturing process cleaning and removing the support material that fills the empty areas. Section SC (Supporting Information) A provides a detailed schematic of the RVE in Figure S2 (Supporting Information) with labels of the relevant geometric parameters and provides the prescribed values for each of the three samples designed in Table S2 (Supporting Information). 1D Capsule Samples 1 and 2 are designed to have the same initial elastic modulus with different critical deformation δ* by varying the initial gap, while 1D Capsule Samples 1 and 3 are designed to have the same critical deformation δ*, and different initial moduli by varying the soft phase radius. An additional sample is manufactured utilizing mechanism A and the experimental results and comparison to Samples 1–3 are provided in Section SC (Supporting Information) to demonstrate an additional way that this bi‐modulus behavior can be achieved.

Both the SolidWorks models and experimental sample images of the upper cap of each sample are shown in Figure 5e. Three cycles of loading for each sample are shown in the cyclic force‐displacement curves in Figure 5f, comparing different δ* (Samples 1 and 2) and different *E*_1_ (Samples 1 and 3), and confirm the desired bi‐modulus behavior and tunability of the designs.

The designed vs. experimentally determined critical displacements for tension, compression, and the total range of displacement before switching state on either end (the summation of the critical values for tension and compression) are shown in Figure 5g. Experimentally, it is seen that $\delta_{c}^{*}>\delta_{t}^{*}$ despite being designed to be the same. The difference is attributed to the thermal expansion of the soft layer during the manufacturing and cleaning process, as it ends up shifting the caps a small amount, causing the compressive critical displacement magnitude to be larger than the tensile critical displacement magnitude. There is very little difference in the values of critical deformation between cycles for the same sample, with the largest standard deviation of the mean across all three cycles presented here for each sample being 1.56 × 10^−2^ mm.

### 4.2 3D Bi‐Modulus Meta‐Capsule

**Figure 6:**
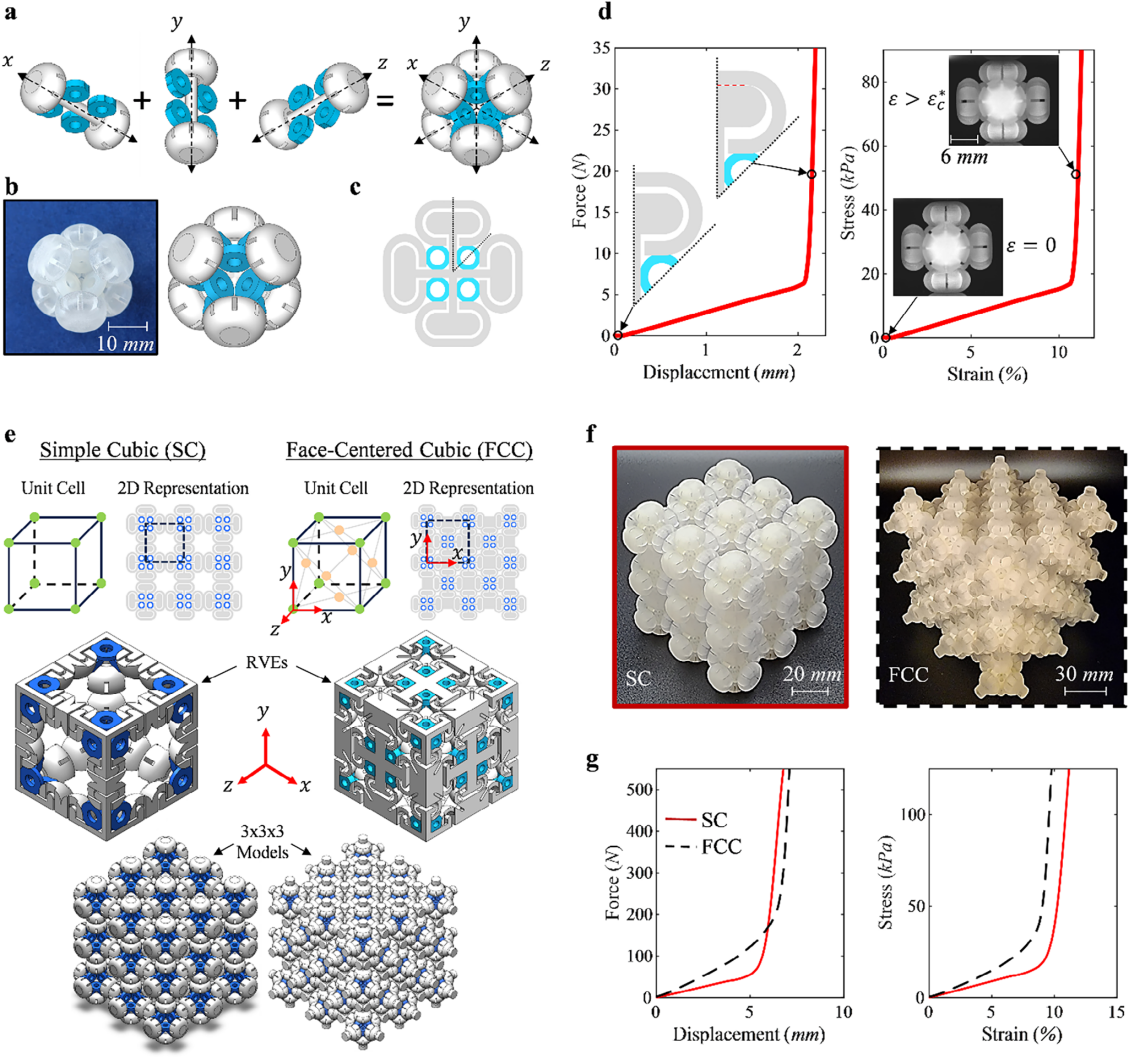
The schematics and experimental results of the bi‐modulus material functioning under both tension and compression in 3D along with two arrangements of its RVE for a bulk material. a) A schematic illustrating the superposition of the 1D bi‐modulus capsule along each axis to obtain the 3D bi‐modulus soft‐ring design where the grey materials in the schematics and SolidWorks models represent the hard phase, and blue material represents the soft phase (colors schemes are consistent throughout the figure), b) a sample image of the soft‐ring design compared with the SolidWorks model, c) a 2D schematical representation of the soft ring design, d) the force‐displacement and stress‐strain response to compressive loading with schematics and experimental images of the initial State 0 and final State 1, e) the two different Bravais lattice structures utilized to make the bulk material of the 3D bi‐modulus material, illustrating each unit cell, 2D representation, RVE, and sample model, f) images of each experimental samples, g) the force‐displacement and stress‐strain response to compressive loading for each sample.

By confirming the bi‐modulus behavior and tunability of the 1D Capsule design under both tension and compression, a 3D Soft‐Ring Capsule design is created and characterized. Previously, it was stated that the soft phase was not required to be a solid layer, so here it was replaced with rings attached to neighboring caps and shafts. The 3D Capsule structure consists of the same 1D Capsule structure superimposed on itself in all three directions as illustrated in **Figure** **6**a. Figure 6b shows the 3D printed Soft‐Ring Capsule and SolidWorks model, and the 2D schematic representation is shown schematically in Figure 6c. The grey regions represent hard phase materials while the blue regions represent soft phase materials. Section SA (Supporting Information) provides a detailed schematic of the design in Figure S3 (Supporting Information) with labels of the relevant geometric parameters, and provides the prescribed geometric values for the sample in Table S3 (Supporting Information).

The soft‐ring design allows for better fine tuning of the initial modulus with adjustment possible to the inner radius, outer radius, and thickness of the rings, whereas the previous solid soft phase design can only be adjusted via its outer radius. With each ring connected directly to neighboring caps, the design is more responsive to multiaxial loading as well. The force‐displacement and effective stress‐strain results are shown in Figure 6d, and experimental images are shown within the stress‐strain curve for both undeformed and after critical deformation where the gap is seen to be closed in the latter. These designs function as bi‐modulus capsules in all three directions under both tension and compression and are just examples of many that can be created using this concept.

### 4.3 3D AMMs with Different Bravais Lattice Structures

With the responses of the I‐Beam, 1D, and 3D Capsuled designs all studied, the next step is to create a “bulk material” with bi‐modulus behavior that is adaptable in all three direction. The 3D Soft‐Ring Capsule is arranged into two different Bravais lattice structures in Figure 6e where the unit cell, 2D representation, RVE, and 3 × 3 × 3 RVE SolidWorks model for the Simple Cubic (SC) unit cell arrangement (left) and for the Face‐Centered Cubic (FCC) unit cell arrangement (right) are all shown. Again, the grey regions represent hard phase materials while the blue regions represent soft phase materials.

The SC arrangement results in each cap being fused directly to only one other cap along the same axis and results in independent bi‐modulus behavior in each direction. The FCC arrangement required the caps of six capsules being fused at each intersection, resulting in a slight alteration in the cap exterior, but not influencing the base capsule geometry, seen in the RVE from the SolidWorks model. This arrangement means that each direction is no longer completely independent from the other directions.

The capsule geometries are identical to the 3D Soft‐Ring Capsule and the two 3 × 3 × 3 RVE arrangements are manufactured for experimentation and shown in Figure 6f. The experimental force‐displacement and effective stress‐strain results for quasistatic compression displayed in Figure 6g show that bi‐modulus behavior is a global behavior of these samples. For the SC arrangement, each individual capsule within the bulk materials reaches their critical deformations at the same time, resulting in a global critical deformation of δ* = 6 mm, as there are three full capsules along the loading direction, each with their individual δ* = 2 mm.

The FCC arrangement results in a complex behavior and shows an ≈2 times stiffer response than the SC arrangement, despite having 4 units (full capsules) in each RVE compared to the SC arrangements 1 capsule. However, the FCC arrangement is not subjecting each capsule to compression due to manufacturing and experimental constraints. The 2D representation schematic for the FCC arrangement in Figure 6e shows how the rows and columns alternate between 2 and 3 capsules each, so for experimental compression the columns with 2 capsules in them are not experiencing the same compression from the boundaries as the column with 3 capsules. This results in FCC utilizing only ≈60% of its capsules while the SC arrangement utilizes 100% of its capsules, making the FCC complex to test and analyze in this early stage of development.

## 5 Conclusion and Discussion

The AMMs discussed here have been proven to function in 1D, 3D, and bulk materials exhibiting both an initial modulus and secondary modulus activating upon reaching a critical deformation. The I‐Beam sample stands as an excellent proof‐of‐concept design to efficiently showcase bi‐modulus behavior in AMMs through experimental, visual, and numerical means. This design also aided in the advance of the FE models, both MM and UMAT, used to further explore adaptive materials. These models both accurately represent the physical I‐Beam sample and allow for direct comparisons of adaptive and non‐adaptive materials, demonstrating how an adaptive material utilizes more material to resist the load while decreasing strain concentrations from the load compared to non‐adaptive materials. In doing so, the material surrounding the applied load is both stiffened and strengthened without sacrificing the compliance and flexibility of the surrounding materials.

The AMMs are also highly tunable as demonstrated with the 1D Capsule designs, so their initial modulus, secondary modulus and critical deformation can all be adjusted, providing an AMM best suited for a certain range of applications and loadings, or to get maximum use out of each material phase by imposing a limit on its deformation to ensure failure in the soft phase will never occur before the hard phase. In addition, the adaptability functions in the elastic regime, meaning that the deformation and adaptations are all reversible and the material can go back to its softer and more compliant state upon unloading. Similar to how 4D printing adds the potential for a material to have a specific additional configuration post manufacturing as a result of a specific external stimuli, the materials discussed here can reprogram itself into potentially limitless new configurations for everchanging external loads, coining the term “infinity‐D” printing.

The 1D and 3D Capsule designs discussed are capable of bi‐modulus behavior under both tension and compression, and the 3D Capsules and SC and FCC arrangements are a small few of many AMMs that can be created that will have adaptive behavior in all three directions. Different Bravais lattice arrangements are also possible and worth exploring, however the current scale and method of sample manufacturing does not allow for large enough samples to be created and tested.

The AMMs described create a synergistic relation between each phase allowing it to achieve a wide range of responses under global and local loading. By introducing additional phases and critical deformation into the design, one can obtain higher orders of additional moduli beyond the secondary for enhanced properties. The responses of AMMs under dynamic loads are another worthwhile direction of exploration.

## 6 Experimental Section

### Mechanical Experiments

Additive manufacturing was used to create all samples using the Stratasys Objet Connex3 multi‐material 3D printer. The hard phases were comprised of either VeroWhite or Vero Clear, while the soft phases were comprised of a softer rubber‐like material, Tango Plus. All bi‐modulus indicators were printed using Agilus Black. The samples were encased in a supporting material after the manufacturing process had been completed, which was removed by hand where possible. The samples were then soaked in the Clean Station to dissolve away any remaining supporting material in areas unable to be cleaned manually.

All mechanical experimentation were performed using the Instron 6800 Series Universal Testing System at quasistatic strain rates of 0.001 1/s. All cyclic loading samples were subjected to three cycles of loading‐unloading, and all compression testing (performed on the 3D bi‐modulus designs) were subjected to only one set of compressive loading. As there were many sample designs created here, the individual dimensions have been specified in the text.

### FE Simulations

Finite element models were created using ABAQUS to simulate and compare to the experimental results and to further examine the benefits of bi‐modulus material for larger material systems. The modeled microstructure's geometry was the same as the initial compression I‐Beam design, where the hard phase was modeled as a linear elastic material with a Young's Modulus of 1.8 GPa with a Poisson's ratio of 0.4, and the soft phase was modeled using a hyperelastic Arruda‐Boyce material model with an initial shear modulus of 0.2495 MPa, a limiting network stretch of 1.6, and an incompressibility parameter of 0.111. The bottom surface was simply supported while the top surface was subjected to a uniaxial compressive displacement of 3.9 mm. Both phases were comprised of CPS4R 4‐node bilinear plane stress quadrilateral, reduced integration, hourglass control elements.

Self‐contact was prescribed for the entire model, and the interaction properties were defined with an out‐of‐plane thickness of 15 mm and frictionless tangential behavior. To better capture the realistic contact of the sample, where the force gradually increases as the sample fully contacts with itself, an interaction property for normal behavior with an exponential pressure‐overclosure was used. Through an iterative process the ideal parameters to replicate the experimental compression sample were determined to be an exponential fit between the two points having a pressure of 0.02 MPa at a clearance of 0 mm, and a pressure of 0 MPa at a clearance of 0.03 mm. The initial and secondary elastic moduli were determined to be 2.4 and 300 MPa respectively and these values were used for the All State 0, All State 1, Auto‐Adapted, and the Layered simulation indentation.

A description of the UMAT model can be found in Section SB (Supporting Information).

## Conflict of Interest

The authors declare no conflict of interest.

## Author Contributions

Y. L. initiated the concept. R. N. and Y. L. designed the experiments and simulations. R. N. conducted experiments, finite element, and analytical analyses. R. N. and Y. L. interpreted the results. Y. L. supervised the overall research. Both authors contributed to the writing of the manuscript.

## Supporting information



Supporting Information

Supplemental Video 1

Supplemental Video 2

Supplemental Video 3

Supplemental Video 4

Supplemental Video 5

## Data Availability

The data that support the findings of this study are available in the supplementary material of this article.
