# Supplementary material for: Adaptive Mechanical Metamaterials with On‐Demand Binary Local Modulus for Embodied Intelligence
Source: Adv Sci (Weinh). 2025 Aug 26;12(40):e09675. doi: 10.1002/advs.202509675 (PMC12561258; doi:10.1002/advs.202509675)
Supplement: Supplementary file 1 — Supporting Information [file ADVS-12-e09675-s001.pdf]

Supplementary Material

Adaptive Mechanical Metamaterials with On-Demand Binary Local Modulus for Embodied Intelligence

Richard J. Nash[a], Yunzheng Yang[a], and Yaning Li[a,1]

[a]Department of Mechanical and Industrial Engineering

Northeastern University, Boston, MA 02215, USA

[1]Corresponding author, E-mail address: y.li@northeastern.edu

Section A: Adaptive Mechanical Metamaterial Designs

A.1 Determining Critical Strain

Hard phase regions are depicted with grey while soft phase regions are depicted with blue. Although it is not necessary for each design to have the same critical deformations for both tension and compression, they have been made equal for simplicity. The critical displacement for each design is obtained by a summation of the corresponding gaps in that given direction shown in Eq. (S1) and Eq. (S2), although again they are equivalent here, so the more general form is used in Eq. (S3).

$$\delta_c^* = 2g_c \quad \text{(S1)}$$

$$\delta_t^* = 2g_t \quad \text{(S2)}$$

$$\delta^* = 2g \quad \text{(S3)}$$

The critical strain is obtained from Eq. (S4), where H is the height of the RVE. As H is easily altered without effecting the critical displacement, the critical strain is arbitrary, and the focus here is on the critical displacement.

$$\varepsilon^* = \delta^*/H \quad \text{(S4)}$$

A.2 I-Beam Design

Fig. S1 shows the schematic of the I-Beam design RVE on the left and a close up of a quarter of the RVE on the right, containing mirror symmetry on each edge, where the relevant geometric

parameters are labeled. Table S1 displays the values of theses relevant geometric parameters used. As shown from the schematics, each "I" is spaced vertically with initial gaps of g_c and g_t between the head of one "I" and the bottom of the neighboring "I". The soft phase connecting each "I" has a thickness of t, and a height of h, both of which can be varied to adjust the initial modulus of the material. For compressive deformation, when gap g_c goes to 0, neighboring hard phases touch over a contact area of A_c, indicated by the red dashed lines.

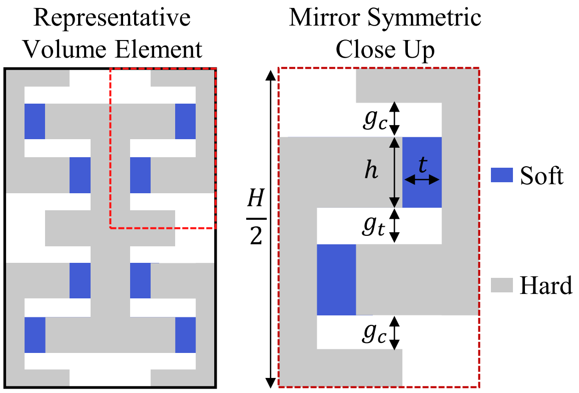

Fig. S1: A schematic of the I-Beam Design with a full RVE shown on the left and a quarter RVE on the right. (The relevant geometric parameters are shown in the quarter model where the quarter model is mirror symmetric about each of its edges. The grey regions represent hard phase materials while the blue regions represent soft phase materials.)

Table S1: The values of the relevant geometric parameters of the RVE, along with the critical deformation that describes the bi-modulus material behavior.

**I-Beam**	H	h	t	$g_c = g_t$	δ^*	ε^*
RVE	15	1.75	0.80	0.75	1.50	0.10

**I-Beam**	H (mm)	h (mm)	t (mm)	$g_c = g_t$ (mm)	δ^* (mm)	ε^*
RVE	15	1.75	0.80	0.75	1.50	0.10

A.3 1D Capsule Design

Fig. S2 shows the schematic of the 1D Capsule Design RVE, with the grips omitted, on the left and a close up of a quarter of the RVE containing mirror symmetry on each edge on the right, where the relevant geometric parameters are labeled, and their values are shown in Table S1. As shown from the schematics, the "I" shaft is completely encompassed by, but not touching, the cap, with initial gaps of g_c and g_t for compression and tension respectively. The top of the shaft has a thickness of h and the cap has a thickness of t. The shaft has radius r_s and the soft phase connecting each cap to the shaft has a radius of R.

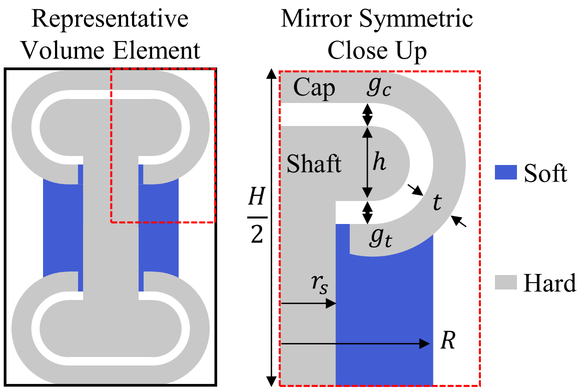

Fig. S2: A schematic of the 1D Capsule Design with the full RVE shown on the left (minus the mechanical grips) and a quarter RVE on the right. (The relevant geometric parameters are shown in the quarter model where it is mirror symmetric about each of its edges. The grey regions represent hard phase materials while the blue regions represent soft phase materials.

Table S2: The values of the relevant geometric parameters of Samples 1-3 of the 1D Capsule Design, along with the critical deformations describing the bi-modulus material behavior.

**1D Capsule RVE**	H	r_s	R	h	t	$g_c = g_t$	δ^*	ε^*
Sample 1	39.5	3	8	3	3	1.5	3	0.076
Sample 2	39.5	3	8	4.5	3	0.75	1.5	0.038
Sample 3	39.5	3	9.5	3	3	1.5	3	0.076

1D Capsule RVE	H (mm)	r_s (mm)	R (mm)	h (mm)	t (mm)	$g_c = g_t$ (mm)	δ^* (mm)	ε^* (mm)
Sample 1	39.5	3	8	3	3	1.5	3	0.076
Sample 2	39.5	3	8	4.5	3	0.75	1.5	0.038
Sample 3	39.5	3	9.5	3	3	1.5	3	0.076

A.4 3D Capsule Design

Fig. S3 shows a 2D representation of the schematic of the 3D Capsule Design, Soft-Ring, meaning the third dimension is omitted for simplification but is present in the actual design as depicted in Fig. 6a. The RVE is shown on the left and a close up of one-eighth of the RVE containing mirror symmetry on each edge is shown on the right. Here the geometric parameters are the same as the 1D Capsule design, now with the addition of the inner and outer soft-ring radii, r_i and r_o, respectively. The out of plane thickness that each ring has been extruded is defined as t_r. The values of the geometric parameters of the RVE are shown in Table S3. This RVE is the same one used for both the single 3D Capsule experiment along with both the SC and FCC arrangement experiments.

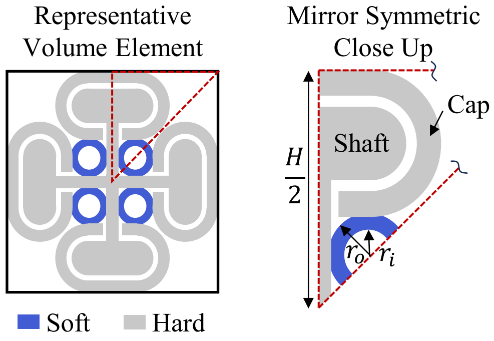

Fig. S3: A 2D representative schematic of the 3D Capsule Design with the full RVE shown on the left and one-eighth of the RVE on the right. (The relevant geometric parameters are the same as the 1D Capsule now with the addition of inner and outer soft ring radius. The one-eighth schematic is mirror symmetric about each of its edges. The grey regions represent hard phase materials while the blue regions represent soft phase materials.)

Table S3: The values of the relevant geometric parameters of the 3D Capsule Design, along with the critical deformations describing the bi-modulus material behavior.

**3D Capsule RVE**	H	r_s	r_i	r_o	r_t	h	t	$g_c = g_t$	δ^*	ε^*
Soft-Ring	19.4	1.5	0.80	1.92	3.0	1.2	1	1.0	2	0.10

**3D Capsule RVE**	H (mm)	r_s (mm)	r_i (mm)	r_o (mm)	r_t (mm)	h (mm)	t (mm)	$g_c = g_t$ (mm)	δ^* (mm)	ε^*
Soft-Ring	19.4	1.5	0.80	1.92	3.0	1.2	1	1.0	2	0.10

Section B: User Defined Material (UMAT) Model

A user defined material (UMAT) was developed for ABAQUS which can simulate “material transfer” during the finite element method simulation, virtually simulating the changing of elements from State 0 to State 1 without having to model the complex and computationally expensive microstructure of a bi-modulus material. This UMAT utilizes an orthotropic plane stress material model with a stiffness matrix shown in Eq. (S5), where E_x is Young’s Modulus along the horizontal direction, E_y is young’s modulus along the vertical direction, ν is the Poisson’s ratio, and G is the shear modulus.

$$\boldsymbol{DDSDDE} = \begin{bmatrix} \frac{E_x^2}{(E_x - E_y\nu^2)} & \frac{E_xE_y\nu}{(E_x - E_y\nu^2)} & 0 \\ \frac{E_xE_y\nu}{(E_x - E_y\nu^2)} & \frac{E_xE_y}{(E_x - E_y\nu^2)} & 0 \\ 0 & 0 & G \end{bmatrix} \quad \text{(S5)}$$

The UMAT code checks the strain in all elements for each time step, and those elements which reach the critical strain are switched from State 0 to State 1 before the next step time. Due to the mixed stress states observed in the simulations with stress concentrations, two individual criteria are in place to trigger the change of states, one for compression and the other for shear, in which the elements changes state upon reaching the prescribed critical strain, ε_y^*, or critical shear strain, γ^*, respectively. The code requires 8 input parameters to fully define the bi-modulus behavior of the I-Beam Design, the critical criteria ε_y^* and γ^*, the unchanging Young’s Modulus in the

horizontal direction E_x, the unchanging Poisson's ratio ν, and the state specific Moduli for the vertical direction, E_1 and E_2, and for shear, G_1 and G_2. The relations of these to the stiffness matrix are described below for reaching the critical compressive criteria:

$$\begin{cases} G = G_1, & \varepsilon_y \\ E_y = E_1, & \varepsilon_y < \varepsilon_y^* \\ E_y = E_2, & \varepsilon_y \geq \varepsilon_y^* \end{cases} \tag{S6}$$

And for reaching the critical shear criteria:

$$\begin{cases} E_y = E_1, & \gamma \\ G = G_1, & \gamma < \gamma^* \\ G = G_2, & \gamma \geq \gamma^* \end{cases} \tag{S7}$$

From simulations of the RVE of the I-Beam Design with periodic boundary conditions the effective material properties have been obtained for use in the UMAT code and are listed below in **Table S4.**

Table S4: The values used for the 8 parameters needed for the UMAT code.

UMAT Parameters	ε_y^*	γ^*	ν	E_x (MPa)	E_1 (MPa)	E_2 (MPa)	G_1 (MPa)	G_2 (MPa)
I-Beam Values	0.1	0.0945	0.0011	3.5	2.4	300	1.5	12.5

Section C: Additional One-Dimensional AMM Design

To further illustrate how the geometry of the soft phase can widely vary while still achieving bi-modulus behavior, another bi-modulus capsule is designed utilizing the shearing soft phase depicted be Mechanism A. Fig. S4a shows this new design utilizing Mechanism A in which the shearing soft phase is only present directly between the cap and shaft of the design, while Fig. S4b shows the original design utilizing Mechanism B.

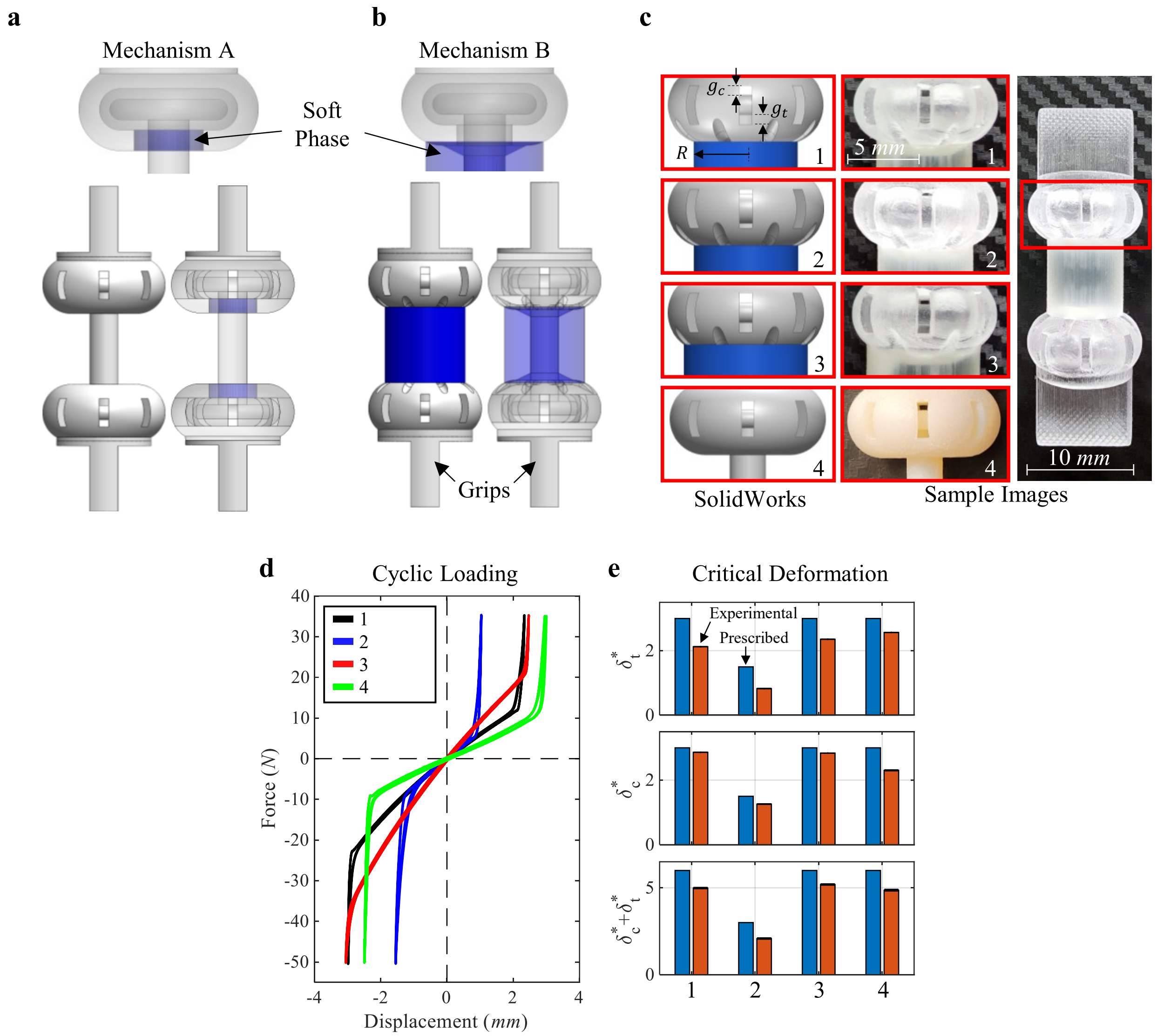

Fig. S4: A comparison of the 1D Capsule Designs utilizing both Mechanisms A and B. a) The relevant geometric parameters are shown in the quarter model where it is mirror symmetric about each of its edges. The grey regions represent hard phase materials while the blue regions represent soft phase materials.
